# Critical Incidents during Anesthesia and Early Post-Anesthetic Period: A Descriptive Cross-sectional Study

**DOI:** 10.31729/jnma.4821

**Published:** 2020-04-30

**Authors:** Binod Gautam, Babu Raja Shrestha

**Affiliations:** 1 Department of Anesthesia and Intensive Care, Kathmandu Medical College, Sinamangal, Kathmandu, Nepal

**Keywords:** *anesthesia*, *critical incidents*, *incident reporting*, *patient safety*

## Abstract

**Introduction:**

Critical incidents related to peri-operative anesthesia carry a risk of unwanted patient outcomes. Studying those helps detect problems, which is crucial in minimizing their recurrence. We aimed to identify the frequency of peri-anesthetic critical incidents.

**Methods:**

This is a hospital-based descriptive cross-sectional study of voluntarily reported incidents, which occurred during anesthesia or following 24 hours among patients subjected to non-cardiac surgery within the calendar year 2019. Patient characteristics, anesthesia, and surgery types, category, context, and outcome of incidents were recorded in an indigenously designed form. Incidents were assigned to attributable (patient, anesthesia or surgery) factor, and were analyzed for the system, equipment or human error contribution.

**Results:**

Altogether 464 reports were studied, which consisted of 524 incidents. Cardiovascular category comprised of 345 (65.8%) incidents. Incidents occurred in 433 (93%) otherwise healthy patients and during 258 (55.6%) spinal anesthetics. Obstetric surgery was involved in 179 (38.6%) incidents. Elective surgery and anesthesia maintenance phase included the context in 293 (63%) and 378 (72%) incidents respectively. Majority incidents 364 (69.5%) were anesthesia-attributable, with system and human error contribution in 196 (53.8%) and 152 (41.7%) cases respectively. All recovered fully except for 25 cases of mortality, which were mostly associated with patient factors, surgical urgency, and general anesthesia.

**Conclusions:**

Critical incidents occur even in low-risk patients during anesthesia delivery. Patient factors and emergency surgery contribute to the most serious incidents.

## INTRODUCTION

A critical incident is any preventable mishap associated with anesthesia administration which leads to or could have led to an undesirable patient outcome.^1^ Underlying causes are diverse, owing to unpredictable interplay amid patient factors, surgical procedure, and effects of anesthetic techniques and drugs. A substantive portion of anesthetic risk may be related to a system or human error.^2^

The critical incident investigation was first used among military pilots aiming to improve their performance and safety.^3^ Cooper and colleagues applied the principle similarly in anesthesia.^4^ Studying critical incidents aids formulate strategies to prevent their recurrence. This might improve anesthesia care and patient safety.^5,6^ Not only developed countries but developing countries have adopted a system to monitor and report anesthesia-related incidents.^7–11^ However, no such initiatives are evident in our country.

The objective of this study was to identify the frequency of peri-anesthetic critical incidents occurring in patients subjected to non-cardiac surgery.

## METHODS

This is a descriptive cross-sectional study conducted over one year from January to December 2019 in the operating rooms, post-anesthesia care unit (PACU), and surgical intensive care unit (ICU) of a teaching hospital. Ethical clearance was obtained from the Institutional Review Committee of the college (Ref: 231120187). All patients who presented for non-cardiac surgery during one calendar year comprised the study population. Patients provided written informed consent for anesthesia and surgery, but a separate consent was not enquired for.

Critical incidents occurring during anesthesia and the following 24 postoperative hours were studied. All incidents were included, such that any adverse event or negative patient outcome (ranging from disturbed bodily function, cardiac arrest to death or canceled surgery) and near-miss (serious error or mishap that had the potential to cause an adverse event but failed to do so because of chance or because it was intercepted) were considered for reporting.

An indigenously designed form was used as a study tool (Appendix). The forms were made available at each study site. All the MD anesthesiology residents were briefed on the objectives and working definitions utilized for the study. They were encouraged and reminded regularly to report incidents that they witnessed, within 24 hours of their occurrence voluntarily. The promise of anonymity and confidentiality was emphasized and they were reassured that no punitive actions would be taken. The filled forms were to be placed in a dedicated file for collection by one of the authors. To minimize missing data and duplication, each report was matched with the patient's details from the census prepared by the respective nurse in-charge and collected each morning from all study sites.

Each single-paged form consisted of spaces to record the incident, its category and patient outcome (recovery, cardiac arrest with recovery, or mortality). Patient demographics, American Society of Anesthesiologists (ASA) physical status score, and existing systemic disease were also part of the form. Surgery characteristics included its specialty and urgency (elective or emergency). Anesthesia information comprised of its type and supervision by consultants. Contextual information included the time of day, location, phase of anesthesia, and means of detection when the incident occurred.

Category of incidents included: airway-intubation, cardiovascular, respiratory, venous access, drug errors, regional anesthesia, equipment-oxygen-power failure and others. Tracheal intubation, vascular access, and regional anesthetic techniques were defined as difficult when the procedure required more than three attempts. Desaturation was defined for arterial oxygen saturation less than 90% and hypothermia for body temperature of less than 35°C. Cardiovascular incidents including bradycardia, tachycardia, arrhythmia, hypotension, and hypertension were defined based on whether corrective medications were administered (clinical significance) as per anesthesia provider's decision.

Data was processed with statistical package for social sciences version 20. Only the anonymously submitted forms with compulsorily filled fields for the incident and its category, primary anesthesia type, and surgical specialty and urgency were reviewed. The analysis of reports was based on a consensus between the authors. If more than one category was possible for one report, the most appropriate or most severe category was chosen. If different incidents occurred during one anesthetic procedure, these were designated as separate incidents. For a co-existent and sequential chain of events, the most severe manifestation of the incident was considered for analysis. Each incident was assigned to an attributable factor: patient, surgery, anesthesia or indeterminate. Anesthesia-attributable incidents were analyzed for potential contributing factors including equipment, system (a set of interdependent elements including people, processes, equipment that interact to achieve a common aim) or human error (lack of knowledge, skill, experience, or judgment and failure to check). Factors associated with mortality occurring within the study period were enlisted.

Descriptive statistics were presented as median (range) for reported patients' age and number (percentage) for categorical variables; and, sub-group comparisons were performed. Relative risks with 95% confidence interval (CI) were calculated for surgical urgency and anesthesia type concerning the occurrence of incidents.

## RESULTS

Among 8012 consecutive patients, 6372 (79.53%) appeared for elective surgery. Within the study duration, a total of 472 filled reports were collected. Eight reports were excluded; six for involving blood-products transfusion without any incident and two for duplication. For analysis, 464 reports were included, which revealed 524 incidents (incident reporting rate of 6.54%).

Patient characteristics and features of anesthesia for the reports are shown in (Table 1).

**Table 1:** Patient and anesthesia characteristics for incident reports.

Variable	Sub-group	Value n (%)
Age		Median: 32 years Range: 9 months to 86 years
Gender	Male	155 (33.4)
	Female	309 (66.6)
Pre-anesthetic evaluation	Done	425 (91.6)
	Not done	28 (6)
	Not mentioned	11 (2.4)
American Society of Anaesthesiologists physical status score	I	151 (32.5)
	II	282 (60.8)
	III	17 (3.7)
	IV	8 (1.7)
	V	6 (1.3)
Co-existing disease	Yes	118 (25.4)
	No	346 (74.6)
Preoperative clinical optimization	Done	253 (54.5)
	Not done	188 (40.5)
	Not mentioned	23 (4.9%)
The primary mode of anesthesia	General	145 (31.3)
	Spinal	258 (55.6)
	Epidural	4 (0.9)
	Combined spinal-epidural	7 (1.5)
	Regional	16 (3.4)
	General + Regional	29 (6.3)
	Intravenous sedation	3 (0.6)
	Local (field)	2 (0.4)
Anesthetic conversion to general anesthesia		31 (6.68)

Incidents occurred more frequently with spinal anesthesia (SA) compared to general anesthesia (GA). With the yearly anesthesia workload of SA in 2849 and GA in 2503 patients (excluding GA plus regional anesthesia, and intravenous sedation), the relative risk was 1.51 (95% CI: 1.24 to 1.84).

The distribution of reports by surgical specialty is shown (Table 2).

**Table 2:** Surgical specialty for incident reports.

Surgical specialty	Incident reports n (%)
General surgery	129 (27.8)
Obstetrics	179 (38.6)
Gynaecology	22 (4.7)
Orthopaedics	58 (12.5)
Ear-Nose-Throat	20 (4.3)
Urosurgery	18 (3.9)
Neurosurgery	20 (4.3)
Plastic-Reconstructive	12 (2.6)
Dental-Maxillo-Facial	5 (1.1)
Thoracic	1 (0.2)

Incidents were most common in obstetric followed by general surgical patients; whereas, the maximal yearly surgical workload comprised of 1995 (24.4%) for general surgery followed by 1214 (15.15%) cases for obstetric surgery. The majority 293 (63.1 %) of incidents involved elective surgery as compared to emergency surgery 171 (36.9%), with the calculated relative risk of 2.14 (95% CI:1.79 to 2.57).

The occurrence of incidents according to location and phase of anesthesia are shown (Table 3).

**Table 3:** Location and phase of anesthesia for incident occurrence.

Location and phase of anesthesia	n (%)
Operating room	493 (94.26)
Anesthesia induction	97 (18.5)
Anesthesia maintenance	378 (72.1)
Emergence-recovery	19 (3.6)
Post-anesthesia care unit	19 (3.6)
Surgical intensive care unit	11 (2.1)

Most incidents occurred during the maintenance phase of anesthesia. The majority of them occurred during regular working hours 465 (88.7%). Means for detection of incidents included equipment monitoring in 217 (41.4%), anesthesiologist's clinical examination in 148 (28.2%), their combinations in 153 (29.2%), and nurses in six (1.1%) of cases. For patients in whom incidents occurred in the operating room, anaesthesiologist's supervision was affirmative in 340 (77.44%) cases, whereas primary anesthesia provider included supervised trainee-resident in 239 (64.94%), lecturer in 53 (14.4%), consultant in 56 (15.21%) and professor in 20 (5.43%) cases.

Reported incidents and their categories are shown (Table 4).

**Table 4:** Category and type of incidents.

Category	Incident n (%)
	Difficult intubation: 14 (2.7)
	Esophageal intubation: 2 (0.4)
	Endo-bronchial intubation: 1 (0.2)
Respiratory: 22 (4.2)	Airway trauma during intubation: 1 (0.2%)
	Endotracheal tube dislodgement: 2 (0.4%)
	Airway obstruction: 5 (1)
	Endotracheal tube cuff leak: 1 (0.2)
	Difficult laryngeal maskinsertion: 2 (0.4%)
	Desaturation: 4 (0.8)
	Bronchospasm: 6 (1.1)
	Laryngospasm: 10 (1.9)
	Aspiration: 2 (0.4)
	Hypotension: 246 (46.9)
	Bradycardia: 51 (9.7)
	Vasovagal reaction during epidural catheterization: 2 (0.4)
	Hypertension: 14 (2.7)
Cardiovascular: 345 (65.8)	Tachycardia: 2 (0.4)
	Cardiac arrestneeding chest compression: 22 (4.2)
	Arrhythmias: 4 (0.8)
	Suspected myocardial infarction: 3 (0.6%)
	Suspected pulmonary embolism: 1 (0.2%)
	Difficult spinal anesthesia: 7 (1.3)
	Failed spinal anesthesia: 21 (4)
	High spinal block: 1 (0.2)
Regional anesthesia technique: 50 (9.5)	Failed regional anesthesia: 14 (2.7)
	Failed epidural anesthesia: 1 (0.2)
	Dural puncture at epidural anesthesia: 2 (0.4)
	Failed combined spinal-epidural anesthesia: 1 (0.2)
	Post-dural puncture headache: 3 (0.6)
	Oxygen supply failure: 2 (0.4)
	Power failure: 7 (1.3)
	Breathing circuit (leak/obstruction): 4 (0.8%)
Equipment-Power and Oxygen supply: 22 (4.2)	Stuck expiratory valve: 2 (0.4)
	Failure to turn on Oxygen: 1 (0.2)
	Vaporizer leak: 1 (0.2)
	Capnograph not functioning: 3 (0.6%)
	NIBP monitor not working: 1 (0.2)
	Laryngoscope malfunction: 1 (0.2%)
	Shivering: 25 (4.8)
Hypothermia/ shivering: 28 (5.3)	Hypothermia: 3 (0.6)
	Wrong drug (syringe swap): 2 (0.4%)
Drug errors: 5 (1)	Drug overdose: 1 (0.2)
	Inadequate effect of non-depolarizing muscle relaxant: 1 (0.2)
	Inadequate effect of reversal agents: 1 (0.2)
	Difficult venous access: 1 (0.2)
Vascular access: 3 (0.6)	Difficult arterial cannulation: 1 (0.2)
	Difficult central vein catheter insertion: 1 (0.2)
	Suspected anaphylaxis: 8 (1.5)
	Delayed recovery: 4 (0.8)
	Hypoglycemia: 2 (0.4)
Others: 21 (4)	Seizure: 2 (0.4)
	Postoperative pain: 2 (0.4)
	Urinary retention: 2 (0.4)
	Anxiety: 1 (0.2)

Values in number (percentage); NIBP: non-invasive blood pressure.

The most common category was cardiovascular followed by regional anesthesia technique-related events.

Complete recovery was achieved from 499 (95. 2%) incidents, among which three patients had recovered from intra-operative cardiac arrest, and intended surgery was canceled in two patients. Twenty-five cases suffered from mortality (3.12 per 1000 anesthetics). Mortalities were found to have the most frequent associations with the general anesthesia (all cases), ASA class greater than two (14 cases), no preoperative clinical optimization (19 cases), emergency nature of surgery (14 cases), and post-anesthetic phase (21 cases) at out of working hours (19 cases) for their occurrence.

## DISCUSSION

This study highlights that with regular briefing and encouragement, reporting on critical incidents associated with anesthesia is feasible even in our low resource set up. The most important findings included cardiovascular events being the incident category, obstetrics as the surgery type, and maintenance phase of anesthesia as the time of incident occurrence. Patient factors, surgical emergency, general anesthesia, and post-anesthetic phase were the most frequent factors associated with mortality.

Despite advances in techniques, medications, equipment, training modalities, and vigilance in care, anesthesia carries a risk of adverse patient outcomes. Although rare, this risk is still reducible. For this purpose, knowledge on the frequency of incident types, their underlying circumstances, and classes of errors at the particular set up is essential. Aiming to initiate a system of reporting and to promote the development of protocols and policies, the present study was planned. Our institution is a tertiary hospital in a city with 700 beds. Under coverage of the Anesthesia department, there are 11 major operating theatres, 16 bedded PACU, and 11 bedded surgical ICU. Providing anesthesia for all procedures except cardiac and transplant surgeries, our department comprises ten anesthesiologists, 12 MD residents, and six medical officers.

The rate of incident reporting (6.5%) in our study was higher than the range of 0.28 to 2.8% reported in the literature for incidents d uring anesthesia. ^9,10,12^ Similar to our finding, a study from Nigeria achieved a rate of 6.1%, but in which obstetric surgeries were not included.^11^ The even higher incident reporting rate of 10% has recently been published from Namibia.^13^ Variation in reporting rates may be affected by the employed definitions, study population, methods for reporting, motivation, and institutional safety culture. Seemingly trivial events like difficult vascular access, anxiety, and post-operative pain were also included in our study, as they were all associated with surges in monitored hemodynamic parameters. Similarly, shivering that occurred exclusively during spinal anesthesia, might bear serious implications apart from patient discomfort, particularly in myocardial ischemiaprone patients and if not corrected in time. We believe that guaranteed anonymity and assurance of no punitive action must have encouraged frank reporting; and, the achievement of far more information because of the inclusion of every possible incident, comprised the major strength of our study. The reports were collected for a calendar year duration aiming to detect more varieties of incidents. In comparison to the reviews of medical charts, prospective studies allow the issuance of timely warnings and advice whenever deemed necessary.^14,15^

In our study, the most common incident category was cardiovascular (65.8%); and, hypotension (46.9%) comprised the majority of incidents. Similar findings were reported previously.^10,16^ A study that excluded obstetric surgeries, observed cardiovascular (41%) as the most frequent incident category.^11^ Whereas another multicentral study comprising 72% of the surgical workload from obstetrics and gynaecology reported a 70% prevalence of hypotension.^13^ Obstetric and gynaecological surgeries comprised 22.7% workload in our practice, second only to general surgery (24.4%). As regional anesthesia (RA) is the technique of choice for cesarean sections and during which universally accepted lower thresholds for defining maternal hypotension must have accounted for the obstetric population and the spinal anesthesia being more frequently associated with the incidents. Even though all of these incidents recovered completely, an in-depth investigation of incidence and risk factors for adverse events in our obstetric population is recommended, to reflect on the ongoing practice, which might suggest a need for change if any.

Our lower rates on airway and respiratory incidents contrast with the studies which observed breathing circuit disconnection or airway-respiratory events as the most frequent incidents.^4,7,12^ Although factors including pre-use checking, and use of monitors and alarms at our set up could have contributed, the most likely reasons for the discrepancy must have been the diversity of reporting methods and differences in nomenclatures employed.

The second-most common category in our study was related to the regional anesthesia technique. In a previous study, the largest group of reported critical incidents were technical difficulties with RA, but compared to ours, with a slightly lesser incidence rate of 40/10000 anesthetics.^16^ Inclusion of neuraxial techniques (spinal and epidural anesthesia) to the same category in our study might have accounted for this discrepancy. More importantly, failure of primary technique and the need for conversion to GA involved 6.68% of reports. Inadequate block and conversion of RA accounted for 10.2% of incidents in a previous study.^16^ Technical difficulties during RA are an inevitable occurrence. However, as the majority of these reports revealed the trainee-residents as the primary anesthesia provider and a substantial proportion mentioned no anesthesiologist's supervision, we propose a thorough study on predisposing factors. Especially for peripheral nerve and plexus blockades, ensuring adequate time interval for local anesthetic onset and a provision of a separate room for performing these procedures may be the first step in tackling the issue, in addition to a proper implementation of ultrasound guidance. To minimize failure rates with RA, the departmental policy might need revision to discourage unsupervised attempts by trainee-residents, together with the provision of a graded skill delivery.

Most incidents occurred during the maintenance phase of anesthesia in our study. This finding has been consistent with numerous studies.^4,7,9,12,17^ Reported incidents were maximum in ASA II patients, probably because a maximum number of patients belonged to the same class in our practice. Whereas, higher ASA class was a frequent factor among mortalities, together with emergency surgery, GA and post-anesthetic period. Emergency surgery and higher ASA classes are already established as the most important predictors of perioperative risk.^12,17,18^ This emphasizes the importance of appropriate timing of surgeries to allow the utmost pre-operative optimization of sicker patients. Also, 19 out of 25 mortalities occurring in patients who were operated during out of regular working hours might indicate that involvement of more than a single anesthesiologist and experienced surgeons during high-risk emergency surgeries may be beneficial.

The most frequent association of mortality with GA in our study must have resulted from a fact that the most high-risk patients and those undergoing high risk procedures such as neurological, thoracic, upperabdominal and trauma surgeries preferably receive GA. The incidence of 24 hours peri-anesthetic mortality (3.12 per 1000 anesthetics) in our study was a bit higher than those of the results in recent publications.^13,18,19^ However, differences in the numerator, the period of investigation, source of data and denominator result in varying rates for peri-anesthetic mortalities among studies and render comparisons less feasible.

The human error being multifactorial and exertion comprising the most important component, trend on residents' working hours might need re-consideration, to avoid their anesthesia administration following long hours of night duties. Certainly, no one is immune to errors, as outlined in the report “to err is human” by Institute of Medicine (US) in 1999.^20^ With the reporting system in place, we believe we can continue to improve clinical care, assimilate safety culture and decrease errors in anesthetic practice.

Achieving such a wide variety of information in a single piece of paper appears cost-effective, especially when persistent efforts during the process are overlooked. The analysis made us confident to put forth our weaknesses, and seek remedies. We have already seen some of our defective and old anesthetic machines, and laryngoscope blades being replaced. Proper labeling of drug syringes, especially those of antibiotics, which are mostly prepared by surgical residents, are further being emphasized. Finally, the characteristics of typical low-medium income countries including unreliable oxygen and electricity supplies, and unavailable spare oxygen cylinders attached to anesthetic machines still need to be addressed.

As a feature of voluntary systems, under-reporting represents the major limitation of our study. Mostly for our lower rates on drug error and related near-miss, hesitancy to reporting is a possibility.^4,21,22^ Reporting bias also cannot be excluded as the tendency to report only the major, unusual and interesting events, and severe complications exist.^23^ This lack of understanding about what should be reported must have been the most important reason for our lower reporting rate from post-anesthetic periods, followed by the provision of attending PACU calls primarily by surgical residents. Another factor may be the absence of designated recovery areas at our set up, such that a tendency exists to transfer patients to PACU only after they are fully recovered in operating table itself, or if not, surgical ICU would be preferred over PACU. Also, the uncertainty about its benefits might act as a barrier to reporting.^6^ In our study design, it was also impossible to validate the information, as reporting was anonymous, and there were chances of missing contextual information for we lacked the electronic anesthesia data recording system. Other limitations included study restriction to 24 hours, no information on day care surgical patients and non-involvement of other members of health-care staff for reporting.

Patient safety is a cause for concern in health-care systems all over the world. As a landmark development, the World Health Organization, in 2005, emphasized voluntary reporting of medical complications, to enhance learning from failures, and to provide future directives.^24^ In resource-limited settings like ours, attention should still be primarily focused on training, attitude, and strict utilization of checklists and protocols to improve patient care, rather than an actual high expenditure. But, for gaining knowledge on weaknesses, and translating it into quality improvement, the incident reporting system should be introduced in the anesthesia department of all teaching and referral hospitals. Starting with mandatory reporting for deaths and severe harms seems most practical. Sequential introduction across surgical wards, whole hospital and at the national level would be appropriate. We believe that our professional organizations like Nepal Medical Association and Society of Anesthesiologists of Nepal ought to take initiatives in conjunction with the government bodies to ensure the environment at local and national levels, for setting minimum standards on scientific record-keeping and reporting systems. However, preserving motivation and breaking the barrier of budget restrictions seem to be real challenges.

## CONCLUSIONS

Despite advances in the provision of monitoring and care, critical incidents occur during peri-anesthetic periods, mostly during the maintenance phase of anesthesia. Cardiovascular incidents occurring during spinal anesthesia in the obstetric population deserve special attention. Patient factors, general anesthesia, and emergency surgery during out of regular working hours comprise important risks for mortality, justifying the appropriate timing of surgery for high-risk patients, and senior support to the trainees during emergencies and high-risk surgeries. This study has highlighted the importance of incident reporting as a component of education and quality improvement in the anesthesia department. However, persistent effort is necessary to motivate the concerned staff so that incident reporting becomes a culture; after all, for the safety of future surgical patients.

## Conflict of Interest

**None.**
